# Sensitivity to CPT-11 of xenografted human colorectal cancers as a function of microsatellite instability and p53 status

**DOI:** 10.1054/bjoc.1999.1019

**Published:** 2000-02

**Authors:** R A Bras-Gonçalves, C Rosty, P Laurent-Puig, P Soulié, B Dutrillaux, M-F Poupon

**Affiliations:** Institut Curie, UMR 147 CNRS-Institut Curie, 26 rue d'Ulm, Paris, 7523 Cédex 051, France; Faculté de Médecine des Saints-Pères, INSERM U 490, 45 rue des Saints-Pères, Paris, 75 Cédex 06, France

**Keywords:** CPT-11, MSI, *p53*, *top1*, human colorectal cancer, *nude* mice

## Abstract

Biological parameters influencing the response of human colorectal cancers (CRCs) to CPT-11, a topoisomerase 1 (top1) inhibitor, were investigated using a panel of nine CRCs xenografted into *nude* mice. CRC xenografts differed in their *p53* status (*wt* or *mut*) and in their microsatellite instability phenotype (MSI^+^when altered). Five CRC xenografts were established from clinical samples. All five had a functional p53, two were MSI^+^and three were MSI^–^. Tumour-bearing *nude* mice were treated intraperitonealy (i.p.) with CPT-11. At 10 mg kg^–1^of CPT-11, four injections at 4-day intervals, four of the five xenografts responded to CPT-11 (growth delay of up to 10 days); the non-responder tumour was MSI^−^. At 40 mg kg^−1^of CPT-11, six injections at 4-day intervals, the five CRCs displayed variable but marked responses with complete regressions. In order to assess the role of p53 status in CPT-11 response, four CRC lines were used. HT29 cell line was MSI^−^/ *Ala273-mutp53*, its subclone HT29A3 being transfected by *wtp53*. LoVo cell line was MSI^+^/ *wtp53*, its subclone X17LoVo dominantly expressed *Ala273-mutp53* after transfection. LoVo tumours (MSI^+^/ *mutp53*) were more sensitive than X17LoVo (MSI^+^/ *mutp53*. HT 29 tumours (MSI^−^*Imutp53*), were refractory to CPT-11 while HT29A3 tumours (MSI^−^/ *wtp53*) were sensitive, showing that *wtp53* improves the drug-response in these MSI^−^tumours. Levels of mRNA expression of *top1*, *fasR*, *TP53* and *mdr1* were semi-quantified by reverse transcription polymerase chain reaction. None of these parameters correlated with CPT-11 response. Taken together, these observations indicate that MSI and p53 alterations could be associated with different CPT-11 sensitivities; MSI phenotype moderately influences the CPT-11 sensitivity, MSI^+^being more sensitive than MSI^−^CRC freshly obtained from patients, *mutp53* status being associated with a poor response to CPT-11. © 2000 Cancer Research Campaign


British Journal of Cancer (2000) **82**(4), 913–923
© 2000 Cancer Research Campaign
DOI: 10.1054/ bjoc.1999.1019, available online at http://www.idealibrary.com on IDEAL®

### Sensitivity to CPT-11 of xenografted human colorectal cancers as a function of microsatellite instability and p53 status

**RA Bras-Gonçalves[1], C Rosty[2], P Laurent-Puig[2], P Soulié[1], B Dutrillaux[1] and M-F Poupon[1]**

[1]Institut Curie, UMR 147 CNRS-Institut Curie, 26 rue d'Ulm, 75231 Paris Cédex 05, France; [2]Faculté de Médecine des Saints-Pères, INSERM U 490, 45 rue des Saints-Pères, Paris 75 Cédex 06, France

**Summary** Biological parameters influencing the response of human colorectal cancers (CRCs) to CPT-11, a topoisomerase 1 (top1) inhibitor, were investigated using a panel of nine CRCs xenografted into *nude* mice. CRC xenografts differed in their *p53* status (*wt* or *mut*) and in their microsatellite instability phenotype ($MSI^+$ when altered). Five CRC xenografts were established from clinical samples. All five had a functional p53, two were $MSI^+$ and three were $MSI^-$. Tumour-bearing *nude* mice were treated intraperitonealy (i.p.) with CPT-11. At 10 mg $kg^{-1}$ of CPT-11, four injections at 4-day intervals, four of the five xenografts responded to CPT-11 (growth delay of up to 10 days); the non-responder tumour was $MSI^-$. At 40 mg $kg^{-1}$ of CPT-11, six injections at 4-day intervals, the five CRCs displayed variable but marked responses with complete regressions. In order to assess the role of p53 status in CPT-11 response, four CRC lines were used. HT29 cell line was $MSI^-$/*Ala273-mutp53*, its subclone HT29A3 being transfected by *wtp53*. LoVo cell line was $MSI^+$/*wtp53*, its subclone X17LoVo dominantly expressed *Ala273-mutp53* after transfection. LoVo tumours ($MSI^+$/*mutp53*) were more sensitive than X17LoVo ($MSI^+$ /*mutp53*. HT 29 tumours ($MSI^-$/*mutp53*), were refractory to CPT-11 while HT29A3 tumours ($MSI^-$/*wtp53*) were sensitive, showing that *wtp53* improves the drug-response in these $MSI^-$ tumours. Levels of mRNA expression of *top1*, *fasR*, *TP53* and *mdr1* were semi-quantified by reverse transcription polymerase chain reaction. None of these parameters correlated with CPT-11 response. Taken together, these observations indicate that MSI and p53 alterations could be associated with different CPT-11 sensitivities; MSI phenotype moderately influences the CPT-11 sensitivity, $MSI^+$ being more sensitive than $MSI^-$ CRC freshly obtained from patients, *mutp53* status being associated with a poor response to CPT-11. © 2000 Cancer Research Campaign

**Keywords**: CPT-11; MSI; *p53*; *top1*; human colorectal cancer; *nude* mice

Human colorectal cancer is the second most frequent cancer in Western countries, and the third leading cause of death by cancer in both sexes (Wilke, 1996). Staging of tumour progression described by Dukes is the main basis for prognosis. For patients with advanced stage, adjuvant chemotherapy with 5-fluorouracil (5-FU) has been shown to decrease recurrence and improve overall survival (Moertel et al, 1990). Depending on 5-FU regimen, 20–30% of hepatic metastases are sensitive to 5-FU associated with leucovorin (de Gramont et al, 1997; Goldberg et al, 1997).

However, the progression of cancer is often associated with a chemoresistance to agents used as the first-line therapy and an effective second-line alternative is presently lacking. Thus, new anticancer drugs are being developed and biological determinants allowing one to predict response to therapy need to be established. CPT-11 (Campto®, Irinotecan) (Kunimoto et al, 1987), a semisynthetic water-soluble derivative of camptothecin (Wall et al, 1966), demonstrated potent anti-tumour activity against experimental colon cancer tumour models in vivo (Houghton et al, 1995) and also in phase I (Armand et al, 1995) and more recently, in phase II trials (Rougier et al, 1997). The anti-tumour activity is exerted by SN38, a metabolite of CPT-11 (Kaneda et al, 1990), which selectively inhibits DNA-topoisomerase1 (top1) (Tanizawa et al, 1994). Cytotoxicity is due to the formation of SN38-stabilized top1 DNA cleavable ternary complexes (Leteurtre et al, 1993), which impede the DNA-religation reaction (Kjeldsen et al, 1992), resulting in DNA single-strand breaks. Stabilized cleavable complexes are rapidly reversible upon lowering of the time of exposure to CPT-11 (Tanizawa et al, 1995). Elevated top1 protein levels increase the frequency of cleavable complexes (Pommier, 1996), thereby increasing DNA damage. As a consequence, DNA single-strand breaks tend to accumulate, and it is most likely that attempts to replicate and/or repair the damaged DNA enhances the formation of DNA double-strand breaks (Vamvakas et al, 1997).

Molecular characterization of genetic alterations in CRC led to the identification of two carcinogenesis pathways. In 85% of cases, CRC display chromosomal instability, allelic losses at 5q, 17p and 18q, p53 mutations are frequent, they are often left-sided and of poor prognosis. The second group (15%) is characterized by genetic instability, with widespread mutations in microsatellite sequences, a right-sided location and a better prognosis. These different pathways of carcinogenesis could account for a differential sensitivity of CRC to CPT-11. Indeed, the *TP53* gene encodes a multifunctional transcription factor that plays a critical role in cellular response after DNA damage, including G1 arrest followed by either DNA repair or apoptosis. Cells defective for p53 have a survival advantage by lacking the apoptotic programme induced by the toxic effects of anticancer drugs (Fan et al, 1994; Ceraline

*Received 15 March 1999*
*Revised 23 August 1999*
*Accepted 25 August 1999*

*Correspondence to:* M-F Poupon et al, 1998). Cells with functional *wtp53* are found to be dramatically more sensitive to many of the commonly used anticancer agents (Fujiwara et al, 1994; Lowe et al, 1993, 1994; McDonald and Brown 1998). In $MSI^+$ tumours, defects in mismatch repair genes could lead to the accumulation of unrepaired or misrepaired DNA during DNA replication, rendering them more sensitive for CPT-11 effect.

Other resistance mechanisms to CPT-11 could also be implicated. Overexpression of the multidrug resistance gene product, P-glycoprotein, encoded by the *mdr1* gene has been incriminated in tumour cell resistance to numerous compounds (Gottesman et al, 1996). Its role in the lack of sensitivity of tumour cells to CPT-11 has been described (Jansen et al, 1998). Two groups have recently shown that colorectal cancers of the colonic mucosa express the *fas* receptor (*fas*R), a cell membrane determinant which triggers the apoptotic cascade (Meterissian et al, 1997; Strater et al, 1997), and that camptothecin treatment activated the *fas*R in CRC cell lines (Shao et al, 1998). CPT-11, like several anticancer drugs (Micheau et al, 1997), might be able to render tumour cells highly susceptible to *fas*R-dependent apoptosis.

Our aim was to search for genetic alterations and biological parameters determining the response of CRCs to CPT-11. First, we evaluated the in vivo response of nine different xenografted CRCs, which differed in their MSI phenotype and their p53 status. Five of the nine xenografts were established from tumours in our laboratory. Four of the nine xenografts were derived from CRC cell lines: $MSI^+$/*wtp53* (LoVo) and its subclone (X17LoVo) transfected with a *mutp53* expression vector; and $MSI^-$/*mutp53* (HT29) and its subclone (HT29A3) transfected with a dominant *wtp53* expression vector. Second, expression of *top1*, the target of CPT-11, of *mdr1* and of *fas*R was investigated in these CRC xenografts.

### MATERIALS AND METHODS

### Human colon cancer tumours and cell lines

Three primary colorectal tumour specimens (TC1, TC7fal and TC71) and two liver metastases (TC118m and TC124m) were obtained from patients. After tumour resection, xenografts were established by subcutaneous (s.c.) implantation into the scapular area of *nude* mice. LoVo and HT29 cell lines were obtained from the American Type Cell Collection (Rockville, MD, USA). The LoVo cell line ($MSI^+$/*wtp53*) was previously transfected with an *Ala273-mutp53* geneticin resistance expression vector, as described (Pocard et al, 1996), producing the X17LoVo subclone which had stable integrated the plasmid. LoVo was arrested in the G1 phase of the cell cycle after 10-Gy irradiation, while no arrest was obtained after irradiation of X17LoVo cells with a mutated p53. The HT29 ($MSI^-$/*mutp53*) cell line was similarly transfected with a *wtp53* expression vector and a subclone HT29A3 was selected using geneticin as described above; HT29A3 had integrated the plasmid in chromosome 12 (12q11–q12), as evidenced by FISH. HT29A3 was arrested in the G1 phase after inhibition of pyrimidine synthesis, as published by Soulié et al (1999), while no arrest was obtained with HT29 parental cells, showing the dominant function of *wtp53* in the HT29A3 cell line. The four cell lines were established as transplantable tumours (s.c. injection of $2 \times 10^6$ cells). A summary of the clinical and biological information about CRCs and cell lines is given in Table 1.

### Mononucleotide repeat microsatellite analysis

The MSI phenotype was determined by multiplex polymerase chain reaction (PCR) amplification of three microsatellite markers: BAT-26, as described (Hoang et al, 1997), BAT-25 and BAT-RII as described (Zhou et al (1998).

### Detection of p53 mutations in CRCs

Characterization of the *TP53* gene was conducted according to the two-step procedure of (Hamelin et al, 1993).

### Mice

Eight- to 10-week old, 25–30 g body weight, swiss *nu*/*nu* male or female mice, bred in the animal facilities of the Curie Institute, Paris, France were used in these assays. The animals were maintained under specified pathogen-free conditions. Their care and housing were in accordance with the institutional guidelines of the French Ethical Committee (Ministère de l'Agriculture et de la Forêt, Direction de la Santé et de la Protection Animale, Paris, France) and under the supervision of authorized investigators.

### Growth inhibition studies

Mice were grafted with tumour fragments of approximately 15 $mm^3$ volume. Mice bearing growing tumours with a volume of 300–500 $mm^3$ were individually identified and randomly assigned to the control or treated group and the treatment started. The animals bearing tumours were sacrificed when their tumour volume reached 2000 $mm^3$, defined as ethical sacrifice. Volumes of individual tumours were calculated from the measurements of two perpendicular diameters using a calliper, performed every 2 days. Each tumour volume (V) was calculated according to the following formula: $V = a^2 \times b/2$, where a and b are the smallest and largest perpendicular tumour diameters. Relative tumour volumes were calculated form the formula: $RTV = (V_X/V_1)$, where $V_X$ = the volume on day x and $V_1$ is the tumour volume at the initiation of therapy (day 1). Growth curves (Figure 1) were obtained for each individual tumour by plotting values of RTV against time (expressed as days after the start of treatment). The anti-tumour activity was evaluated according to four criteria: (i) the growth inhibition (GI), which was calculated according to the following formula: $\% GI = 100-(RTVt/RTVc) \times 100$ where RTVt is the mean RTV of the treated group and RTVc that of the control group, at the time of sacrifice of the first mouse in the group control when it attained a tumour volume of 2000 $mm^3$; (ii) the growth delay was calculated as the time necessary to increase of fivefold the tumour volume of individual mice. The cumulative percentage of individual tumours (mice) reaching this 5×-relative volume increase is plotted as a function of time on Figures 2 and 3. This corresponds to the survival time of mice. Statistical significance of the differences between the individual growth delays was tested by a log-rank test; (iii) a relative growth delay was calculated as the difference between the time in days required for control and treated tumours to reach a fivefold increase in volume (Houghton et al, 1995). A ratio between the growth delays of tumours in the treated group and that of the control group was also calculated and reported on Tables 2 and 3; and then (iv) the number of partial responses or complete tumour regressions (PR and CR); PR was

 © 2000 Cancer Research Campaign

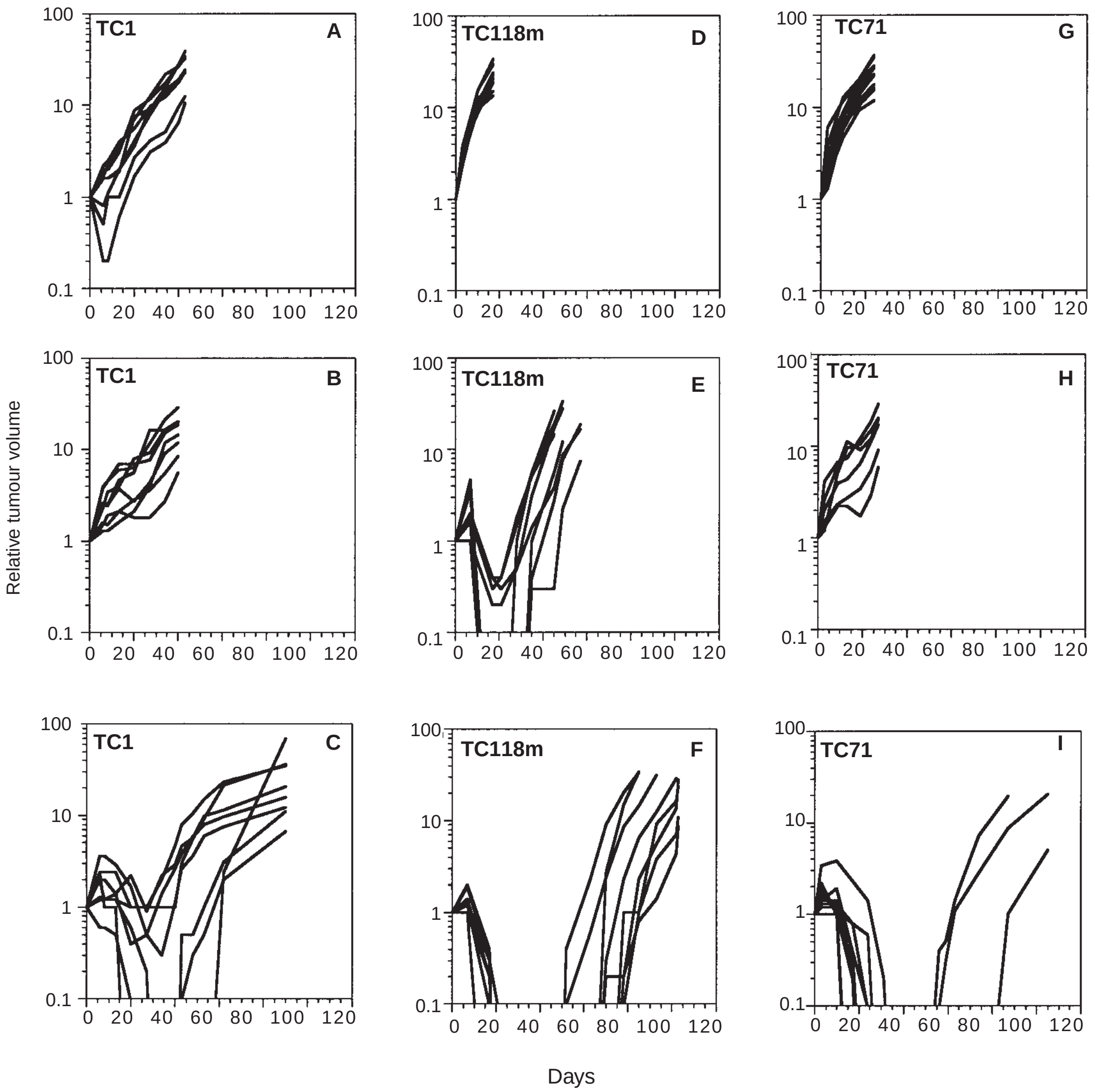

**Figure 1** Anti-tumoural effects of CPT-11 on human colorectal cancers established from clinical samples and subcutaneously grafted into the scapular area of *nude* mice. Individual growth curves for TC1 (**A, B, C**), TC118m (**D, E, F**) and TC71 tumours (**G, H, I**). Tumour-bearing mice were treated with CPT-11 at low dose (**B, E, H**) (10 mg $kg^{-1}$ at 4-day intervals × 4) or high dose (**C, F, I**) (40 mg $kg^{-1}$ at 4-day intervals × 6)

defined as tumour growth inhibition greater than 50% and CR as tumours that had regressed and could no longer be palpated. Mice were considered to be cured if tumour-free after 180 days of observation.

### Formulation and administration

CPT-11 (Campto®, Irinotecan) was generously supplied by Rhône Poulenc Rorer (Vitry sur Seine, France). Stock solution of CPT-11 was diluted in 0.9% sodium chloride solution and administered i.p. in a 0.2 ml volume to tumour-bearing mice using an intermittent schedule: two doses were tested, a high total dose of 240 mg $kg^{-1}$ (6 injections of 40 mg $kg^{-1}$ $day^{-1}$ every 4 days) and a total low dose of 40 mg $kg^{-1}$ (four injections of 10 mg $kg^{-1}$ $day^{-1}$ every 4 days). Mice in the control groups received 0.2 ml of the drug-formulating vehicle with the same schedule as given to the treated animals.

### *top-1, p53, mdr-1and fas-receptor PCR mRNA* expression

Isolation of total RNA from frozen tumour samples weighing approximately 20 mg was done as follows: the frozen tissue was placed under liquid nitrogen and immediately pulverized with a mortar and pestle. The pulverized tissue was rapidly transferred to 1 ml-containing TRIZOL® Reagent (Gibco-BRL, Life Technologies). The resulting RNA pellet was resuspended in 40 µl sterile deionized water, and stored overnight at 4°C for complete dissolution. The concentration of the RNA was determined by spectrophotometry.

Synthesis of complementary DNAs from poly-adenosine (poly(A)) mRNA was done as follows: RNA at a final concentration of 2 µg in 20 µl of sterile water was incubated at 65°C for 10 min. A mix containing 4 µl of 5× RT buffer (Gibco-BRL, Life

© 2000 Cancer Research Campaign

**Table 1** Clinical characteristics of the CRC tumours and cell lines used

| Tumours | Origin | Stage[a] | HNPCC |
|---|---|---|---|
| TC1 | Rectum | B | No |
| TC124m | Liver metastasis | D | No |
| TC118m | Liver metastasis | D | No |
| TC7fal | Right colon | C | No |
| TC71 | Sigmoid | B | Yes[b] |
| LoVo and X17LoVo[c] | Lymph node metastasis | D | No |
| HT-29 and HT29A3[d] | Sigmoid | B | No |

[a]According to Duke's classification. [b]With a familial story of HNPCC.
[c]Subclone of LoVo cells transfected with *Ala273-mutp53* expression vector.
[d]Subclone of HT29 cells transfected with *wtp53* expression vector.

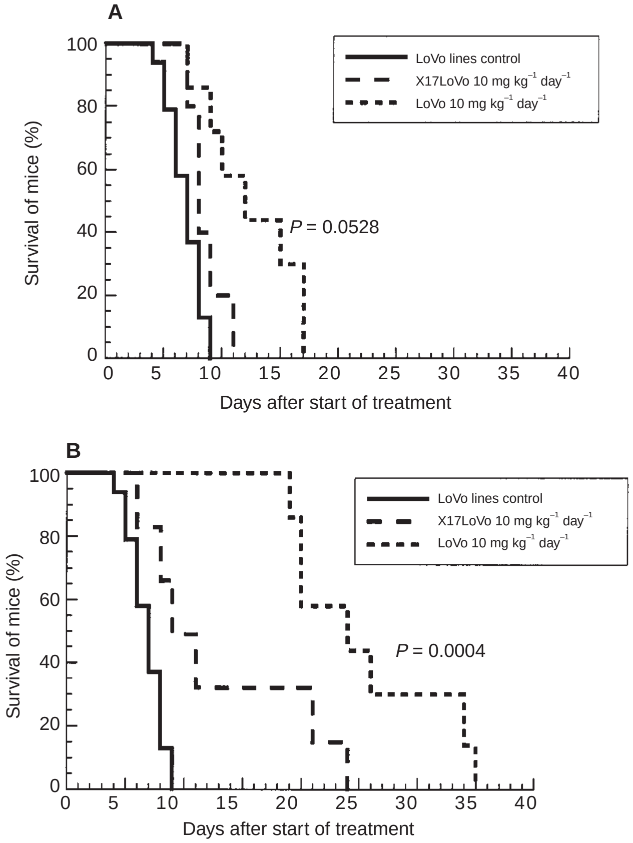

**Figure 2** Effect of CPT-11 on the survival of mice bearing human colorectal cancers established from the cell lines LoVo and X17LoVo, a subclone of LoVo transfected with a dominant *mutp53*. Survival curves are plotted as a function of the survival time of mice, which were sacrificed as their tumour reached 2000 mm$^3$. Mice were treated with CPT-11 at low dose (**A**) (10 mg kg$^{-1}$ at 4-day intervals × 4) or high dose (**B**) (40 mg kg$^{-1}$ at 4-day intervals × 6). *P*-values (log-rank test) corresponding to the differences between the growth curves of CPT-11 treated LoVo and X17LoVo tumours, are indicated

Technologies), 2 µl dithiothreitol (DTT) (100 mM), 1 µl of oligo-dT (200 ng µl$^{-1}$), 1 µl of a mix containing 12.5 mM of each dNTP (final concentration 2.5 mM) and 0.2 µl (200 U µl$^{-1}$) of RNAase H reverse transcriptase (Gibco-BRL, Life Technologies) was added to the RNA and incubated at 45°C for 60 min.

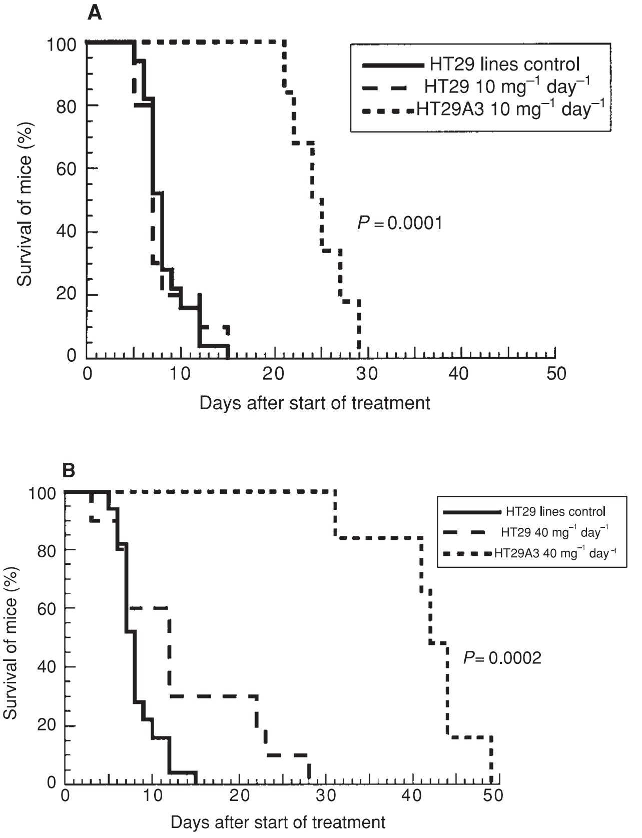

**Figure 3** Effect of CPT-11 on the survival of mice bearing human colorectal cancers established from the cell lines HT29 and HT29A3, a subclone of HT29 transfected with a dominantly expressed *wtp53*. Survival curves are plotted as a function of the survival time of mice, which were sacrificed as their tumour reached 2000 mm$^3$. Mice were treated with CPT-11 at low dose (**A**) (10 mg kg$^{-1}$ day$^{-1}$ at 4-day intervals × 4) or high dose (**B**) (40 mg kg$^{-1}$ at 4-day intervals × 6). *P*-values (log-rank test) corresponding to the differences between the growth curves of CPT-11 treated HT29 and HT29A3 tumours, are indicated

### PCR primers

The primers listed below were selected for their specificity and their selectivity towards human gene sequences (purchased from Oligo Express, Paris).

***top1*-1**: S 5′-AAA AGT CCA AGC ATA GCA ACA G-3′ (bases 345–365 of the topoisomerase-1 gene coding sequence)
***top1*-2**: AS 5′-AGG AAC AAA ATA GCC ATC ATC T-3′ (bases 574–595 of the topoisomerase-1 gene coding sequence)
***mdr1*-1**: S 5′-AGC GCG AGG TCG GGA TGG ATC TTG AAG GGG A-3′ (bases 411–441 of the *mdr*-1 gene coding sequence (Murphy et al, 1990))
***mdr1*-2**: AS 5′-TTG ACA TCA GAT CTT CTA AAT TTC CTG CAT TT-3′ (bases 664–695 of the *mdr*-1 gene coding sequence (Murphy et al, 1990))
***p53*-1**: S 5′-ACA CGC TTC CCT GGA TTG G-3′ (bases 168–186 of the *p53* gene coding sequence (el-Mahdani et al, 1997))
***p53*-2**: AS 5′-GGT CTT GGC CAG TTG GCA A-3′ (bases 616–634 of the *p53* gene coding sequence (el-Mahdani et al, 1997))
***fas* R-1**: S 5′-CAA GTG ACT GAC ATC AAC TCC-3′ (bases

 © 2000 Cancer Research Campaign

**Table 2** p53 status, MSI phenotype and responses to CPT-11 of CRC xenografts in *nude* mice established from clinical samples

| Tumour xenografts | *p53* | MSI | CPT-11 total dosage mg kg$^{-1}$ | Growth rate (days)[a] mean ± | Growth inhibition %[b] (days) | Growth delay in days (ratio)[c] | Nb mice per group | Nb PR[d] | Nb CR[e] | Nb cures[f] |
|---|---|---|---|---|---|---|---|---|---|---|
| TC1 | *wt* | – | 0 | 23 ± 8 | | | 8 | | | |
| | | | 40 | 22 ± 10 | 0 | 0 | 8 | 3 | 0 | 0 |
| | | | 240 | 54 ± 12** | >99 (30) | 31 (2.3) | 8 | 5 | 3 | 0 |
| TC124m | *wt* | – | 0 | 11 ± 3 | | | 6 | | | |
| | | | 40 | 21 ± 4** | >99 (20) | 10 (1.9) | 7 | 7 | 0 | 0 |
| | | | 240 | 31 ± 6** | >99 (20) | 20 (2.8) | 7 | 4 | 3 | 0 |
| TC118m | *wt* | – | 0 | 6 ± 1 | | | 8 | | | |
| | | | 40 | 41 ± 8*** | >99 (17) | 35 (6.8) | 8 | 4 | 4 | 0 |
| | | | 240 | 86 ± 13*** | 100 (17) | 80 (14) | 8 | | 8 | 0 |
| TC7fal | *wt* | + | 0 | 8 ± 3 | | | 8 | | | |
| | | | 40 | 27 ± 3*** | >99 (17) | 19 (3.4) | 9 | 5 | 4 | 0 |
| | | | 240 | 44 ± 6*** | >99 (17) | 36 (5.5) | 9 | 5 | 4 | 0 |
| TC71 | *wt/mut* | + | 0 | 7 ± 2 | | | 10 | | | |
| | | | 40 | 15 ± 9* | >99 (24) | 8 (2.1) | 6 | 6 | 0 | 0 |
| | | | 240 | 86 ± 17*** | 100 (24) | 79 (12.3) | 10 | | 10 | 7 |

[a]Delay in days to reach a fivefold increase of CRC xenograft volume from their initial size at the start of treatment (300–500 mm$^3$). [b]GI: treated/control growth inhibition, calculated at the day (in parentheses) of the ethical sacrifice of the first control mouse bearing a tumour with a volume of 2000 mm$^3$. [c]In parentheses, ratio between growth delays of the tumours in the treated group and that of the controls. [d]Up to 50% of growth inhibition, calculated at the ethical sacrifice of the first control mouse. [e]Complete regressions. [f]Tumour free mice were defined as bearing non palpable tumour after the end of the experiment (180 days). [g]Significantly different from control groups: *$0.01 < P < 0.05$; **$10^{-6} < P < 10^{-4}$; ***$P < 10^{-7}$.

**Table 3** Responses to CPT-11 treatment of CRC cell lines varying for their p53 status and MSI phenotype established as xenografts in *nude* mice

| Tumour xenografts | *p53* | MSI | CPT-11 total dosage mg kg$^{-1}$ | Growth inhibition %[b] (days) | Nb mice per group | Nb PR[d] | Nb CR[e] | Nb cures[f] |
|---|---|---|---|---|---|---|---|---|
| LoVo | *wt* | + | 0 | | 7 | | | |
| | | | 40 | 60 (8) | 7 | 6 | 0 | 0 |
| | | | 240 | 80 (8) | 7 | 7 | | 0 |
| X17LoVo | *wt/mut*[h] | + | 0 | | 7 | | | |
| | | | 40 | 30 (7) | 5 | 2 | 0 | 0 |
| | | | 240 | 50 (7) | 6 | 4 | 0 | 0 |
| HT29 | *mut* | – | 0 | | 10 | | | |
| | | | 40 | 31 (22) | 9 | 2 | 0 | 0 |
| | | | 240 | 66 (22) | 9 | 7 | 0 | 0 |
| HT29A3 | *mut/wt*[h] | – | 0 | | 6 | | | |
| | | | 40 | 70 (20) | 6 | 6 | | 0 |
| | | | 240 | >99 (20) | 6 | 4 | 2 | 0 |

[a]Delay in days to reach a fivefold increase of CRC xenograft volume from their initial size at the start of treatment (300–500 mm$^3$). [b]GI: treated/control growth inhibition, calculated at the day (in parentheses) of the ethical sacrifice of the first control mouse bearing a tumour with a volume of 2000 mm$^3$. [c]In parentheses, ratio between growth delays of the tumours in the treated group and that of the controls. [d]Up to 50% of growth inhibition, calculated at the ethical sacrifice of the first control mouse. [e]Complete regressions. [f]Tumour free mice were defined as bearing non palpable tumour after the end of the experiment (180 days). [g]Significantly different from control groups: *$0.01 < P < 0.05$; **$10^{-6} < P < 10^{-4}$; ***$P < 10^{-7}$. [h]Dominant p53 in transfected lines.

270–290 of the *fas-apo*-1 receptor gene coding sequence (Laurence et al, 1996))
***fas*R-2**: AS 5′-CCT TGG TTT TCC TTT CTG TGC-3′ (bases 797–817 of the *fas-apo*-1 receptor gene coding sequence (Laurence et al, 1996))
***hprt*-1**: S 5′-CAA CAG GGG ACA TAA AAG TAA T-3′ (bases 414–435 of the *hprt*-gene coding sequence)
***hprt*-2**: AS 5′-AGT CCG TCA TAT TAG GTT TCT-3′ (bases 534–554 of the *hprt*-gene coding sequence).

### PCR conditions

The final PCR reaction volume was 50 µl: 1 µl (1/20) of the cDNA solution was mixed with 5 µl of 10× *Taq* buffer (Appligene-Oncor, Illkirch, France), 1 µl dNTP (final concentration 2.5 mM each), 1 µl of each of the 5′ and 3′ primers (100 ng µl$^{-1}$), 40.5 µl water and finally 0.5 µl (2.5 U) *Taq* polymerase (Appligene-Oncor, Illkirch, France) was added. After preheating at 94°C (hot-start), the tubes were placed in a Perkin-Elmer 2400 thermocycler

© 2000 Cancer Research Campaign 

(Yvelines, France) for 3 min at 94°C followed by a maximum of 35 cycles (1 min at 94°C, 1.5 min at 60°C and 2 min at 72°C), and a final elongation at 72°C for 7 min.

### Quantitation of the PCR products

The linear range of amplification for each cDNA was established, according to a kinetic analysis (Köhler et al, 1995); 7 µl of the PCR products were consecutively pipetted at the end of each chosen cycle number within a preliminary chosen range of 10–15 cycles, the final elongation at 72°C for 7 min being performed immediately after, and stored at 4°C. Four microlitres of a gel loading solution (bromophenol blue and xylene cyanol) were added and the solution was electrophoresed on a 1.8% agarose gel stained with ethidium bromide (final concentration of 0.5 µg $ml^{-1}$). After running 50 min at 100V, the gel was washed in distilled water and placed on a UV illuminator (Imager, France) for a 6/30 s exposure, the image was then processed by software NIH image 1.60 version. These conditions were rigorously used in all experiments.

As the amplified PCR product is proportional to the initial amount of DNA only in the linear phase of the amplification reaction, we defined the linear range of expression for each gene. To calculate the relative expression of the genes of interest, a ratio was calculated between the fluorescent signal given by the BET-intercalated PCR product within the linear amplification range of the gene of interest and that given by that of the *hprt* gene.

### RESULTS

### Response to CPT-11 of human colorectal cancers xenografts varying for their MSI phenotype

Five CRC tumours obtained from clinical samples were used and established as xenografts. They originated from the left part of the colon or from the rectum. Their staging differed, stage B, B and C for TC1, TC71 and TC7fal respectively, and stage D for TC124m and TC118m. The TC71 tumour originated from a patient with a clinical history of hereditary non polyposis colon cancer (HNPCC) (Table 1). All had a functional p53, including the TC71 which was heterozygote with a mutation at the codon 176. The p53 status was determined by DGGE for TC118m and TC124m and has been previously described for TC1, TC7fal and TC71 (Hamelin et al, 1993; Cottu et al, 1996). These CRCs differed in their MSI phenotype, three being $MSI^-$ (TC1, TC124m and TC118m) and 2 $MSI^+$ (TC7 and TC71). Microsatellite instability of tumours was measured by band shift of three probes (BAT 25, BAT 26 and BAT RII), as described by Olschwang et al (1997) (Table 4).

CPT-11 at a total dose of 240 mg $kg^{-1}$ (high dosage) given as six i.p. injections of 40 mg $kg^{-1}$ at 4-day intervals was well tolerated. CPT-11 was also given at a total dose of 40 mg $kg^{-1}$ (low dosage) as four injections of 10 mg $kg^{-1}$ at 4-day intervals. The responses of the five CRC tumours established from clinical samples to both CPT-11-treatment protocols are summarized in Table 2. Without treatment, the growth rates of CRC xenografts varied between 6 and 23 days for TC118m and TC1 respectively. Given at a high dose, CPT-11 caused significant growth delays of all CRCs: 31, 20, and 36 days for TC1, TC124m and TC7fal respectively, representing 2.3-, 2.8- and 5.5-fold decreases in growth rate respectively. Impressive growth delays were observed for TC118m and TC71, up to tenfold their control growth rate. Complete regressions produced by CPT-11 at high dosage were observable in all CRC tumour models, with the CR frequency varying from 3/8 to 10/10 for TC1 and TC71 respectively. Cures were observed only in the group of TC71 tumours, with a frequency of 70% (7/10): no palpable tumours at day 180 after start of the treatment and histological examination of the graft site revealed fibrosis tissue and the absence of remaining living tumour cells.

At low dosage, CPT-11 caused a growth delay of 35 days for TC118m, the effect was less marked for TC124m, TC7fal and TC71 and absent for TC1. Complete regressions were observable in TC118m and TC7fal, with the PR frequency varying from 3/8 to 6/6 for TC1 and TC71 respectively. All tumours regrew.

Individual growth curves of TC1, TC118m and TC71 in the control, low and high dose CPT-11 groups are shown in Figure 1. They allow the variability of drug responses in individual mice to be evaluated for the three CRC models. TC1 was the least aggressive of the three CRC, and mice survived 40 days in the control group. CPT-11 at low dose did not modify TC1 tumour progression. At high dose, all eight TC1 tumours were slowed down and three complete regressions were observed. No mice survived beyond day 90 after start of treatment. TC118m grew very rapidly in the control group and no mice survived beyond day 20. All TC118m-bearing mice responded to CPT-11 at low dose, with four complete regressions; at high dose, all had regressed completely at day 20. However, all regrew between days 50 and 80. Their growth rate was similar to that of untreated tumours. The growth rate of TC71 tumours was intermediate, all mice survived 30 days in the control group. The efficacy of CPT-11 at low doses was variable. With high dose CPT-11, complete regressions of all the tumours were induced and seven of ten TC71-bearing mice were cured. No acceleration of growth rate was detected in the relapses. Considering all the parameters of responsiveness, all these p53wt colorectal cancer xenografts respond to CPT-11, with variable sensivity; TC1($MSI^-$) was the least sensitive CRC and TC71 ($MSI^+$) the most sensitive, as it was curable by high dose CPT-11.

### Influence of p53 mutation on the response to CPT-11 of xenografted CRC established from $MSI^+$ cell lines

In order to evaluate the role of p53 mutation in CPT-11 response of CRC xenografts, a $MSI^+$ cell line (LoVo) was transfected with a mutated p53 vector. The LoVo cell line was derived from a lymph node metastasis of a CRC. LoVo cells were $MSI^+$ with a homozygous deletion of hMSH2 (Casares et al, 1995) and had a wild-type p53 (Hamelin et al, 1993). Microsatellite instability of tumours was measured by band shift of three probes (BAT 25, BAT 26 and BAT RII), as described by Olschwang et al (1997) (Table 5). LoVo cells were transfected with an *Ala273-mutp53* geneticin resistance expression vector, as described (Pocard et al, 1996), and one subclone, X17LoVo, was selected and established as a xenograft in *nude* mice.

LoVo and X17LoVo established as s.c. tumours were transplanted into mice for testing their sensitivity to CPT-11. The growth rates of LoVo and X17LoVo were very similar, of 6 and 7 days respectively (data not shown). Given at high dose, CPT-11 caused growth inhibitions of 80% and 50% observed for LoVo and X17LoVo respectively, but neither xenograft showed complete regression with high dose CPT-11. Given at low dose, CPT-11 caused growth inhibitions of 60% and 30% for LoVo and X17LoVo respectively, were observed. No complete regressions

 © 2000 Cancer Research Campaign

**Table 4** MSI phenotype and p53 status of the CRC tumours

| | MSI phenotype | | | *p53* status |
|---|---|---|---|---|
| Tumours | BAT-26 | BAT-RII | BAT-25 | mutated codon |
| TC1 | – | – | – | *wt* |
| TC124m | – | – | – | *wt* |
| TC118m | – | – | – | *wt* |
| TC7fal | Band shift | Band shift | Band shift | *wt* |
| TC71 | Band shift | Band shift | Band shift | 176/heterozygote |

were achieved. Kinetics of the number of tumours (as a cumulative percentage of the total number of mice per group) reaching individually a fivefold increase of their initial volume at the start of the treatment, were reported on Figure 2, as a function of time. This parameter corresponds to the survival time of mice, which were sacrificed as their tumour reached 2000 $mm^3$. This parameter did not differ between the two control groups of non-treated tumours (LoVo and X17LoVo, $P$ = 0.07) and data were pooled. Figure 2 visualizes the loss of sensitivity of X17LoVo tumours, at low dosage (Figure 2A) as well as at high dosage (Figure 2B). Differences between LoVo and X17LoVo were statistically significant, as calculated by the log-rank test ($P$ = 0.05 and $P$ = 0.0004), for low dose and high dose experiments respectively. Taking into account the different parameters of responsiveness to CPT-11, X17LoVo (*mutp53*) tumours were less sensitive than LoVo (*wtp53*) xenografts.

### Influence of wtp53 on the response to CPT-11 of CRC xenografts established from $MSI^-$ cell lines

The HT29 cell line was derived from a sigmoid colon cancer of stage B1. HT29 cells were $MSI^-$ and had a mutated p53 in *Ala* 273 codon, as checked by us and published by Hamelin et al (1993) respectively. Microsatellite instability of tumours was measured as specified (Table 5). HT29 was transfected with a *wtp53* geneticin resistance expression vector, according to the methods described in Pocard et al (1996). A selected subclone, HT29A3, had integrated the plasmid in chromosome 12 (12q11–q12), as evidenced by FISH. After inhibition of DNA synthesis, HT29A3 cells were arrested in G1/S, while HT29 cells did not (data not shown). All cell lines were tumorigenic, with 100% tumour takes in our experimental conditions. The growth rates of HT29 and HT29A3 tumours did not significantly differ (of 8 and 10 days respectively $P$ = 0.25). Given at high dose, CPT-11 caused growth inhibitions of 66 and 100% respectively. None of the HT29 xenografts regressed completely under treatment, whereas two of six HT29A3 achieved complete regression. Given at low dose, CPT-11 caused growth inhibitions of 31% and 70% for HT29 and HT29A3 tumours respectively. No complete regression was achieved. All HT29A3 responded at least partially to the treatment, whereas only two of the nine HT29 tumours were slowed down. Individual growth delays of HT29 tumours as calculated as the percentage of tumours (mice) reaching a fivefold increase of the initial tumour volume at the start of the treatment, were reported on Figure 3. Curves corresponding to the survival of non-treated tumours (HT29 and HT29A3) did not significantly differ ($P$ = 0.25) and data were pooled. Figure 3 visualizes the prolongated survival of mice bearing the HT29A3 tumours as compared to that bearing the HT29 tumours, at low dosage (Figure 3A) as well as at high dosage (Figure 3B). These differences were statistically different, as calculated by the log-rank test ($P$ = 0.0001 and $P$ = 0.0002), for low dose and high dose treatments respectively. Taking into account the different parameters of responsiveness to CPT-11, HT29A3 tumours, *wtp53* transfected, were more sensitive than tumours established from HT29, its parental *mutp53* cell line.

### *top1*, *fasR*, *mdr1* and *p53* gene expression in CRC xenografts

Relative expressions of the *top1*, *fasR*, *mdr1* and *p53* genes were determined with respect to the hypoxanthine phosphoribosyl transferase (*hprt*) gene, chosen as the internal standard. The *hprt* gene was expressed at a constant level among the different specimens (Figure 4). The relative expression of the genes of interest was calculated within the linear amplification range of the gene of interest and that given by that of the *hprt* gene (Figure 5). There was little variation of *top1* gene expression between the different tumours. A fourfold variation of *top1* expression among the different tumour samples was found (Table 3). Expression of *mdr1* showed a variation of greater than 80-fold in CRC xenografts, in the conditions described here. These large variations in *mdr1* gene expression were due to exceptionally high values in primary tumours and very low values in metastatic tumours. *p53* gene expression was very high in X17LoVo mutant tumours, 100-fold higher than its parental LoVo cell line. The level of expression of *p53* varied over a tenfold range among the other tumours. *fasR* expression showed a 20-fold variation. *top1*, *mdr1*, *p53* and *fasR* mRNA levels did not correlate with responsiveness of xenografts to CPT-11 (Table 6).

### DISCUSSION

We have tested here the sensitivity to CPT-11 of a panel of nine human colorectal cancers (CRC) differing with respect to their genetic alterations concerning p53 function and/or DNA repair, hypothesizing that these genetic alterations influence their response to CPT-11. This might thereby orientate towards identification of groups of patients who could benefit from top1 inhibitors. CRC xenografts used here originated either from clinical CRC samples (without any intermediate cell culture) or from CRC cell lines, which have been experimentally modified for p53 status.

Among the xenografts established from clinical samples, two were $MSI^+$ and three were $MSI^-$, all were associated with a functional p53 and their responses to low or high dose CPT-11 were compared. At high doses, differences between the responses of the CRCs were seen: both $MSI^+$ tumours were highly sensitive, one (TC71) being curable in 70% of cases. The three $MSI^-$ CRCs

**Table 5** MSI phenotype and p53 status of the cell lines used

| | MSI phenotype | | | *p53* Status |
|---|---|---|---|---|
| Cell lines | BAT-26 | BAT-RII | BAT-25 | Mutated Codon |
| LoVo | –[a] | Band shift | Band shift | *wt* |
| X17LoVo | –[a] | Band shift | Band shift | 273*mut* transfected |
| HT29 | – | – | – | *Ala273* |
| HT29A3 | – | – | – | *wtp53* transfected |

[a]Homozygous deletion of hMSH2.

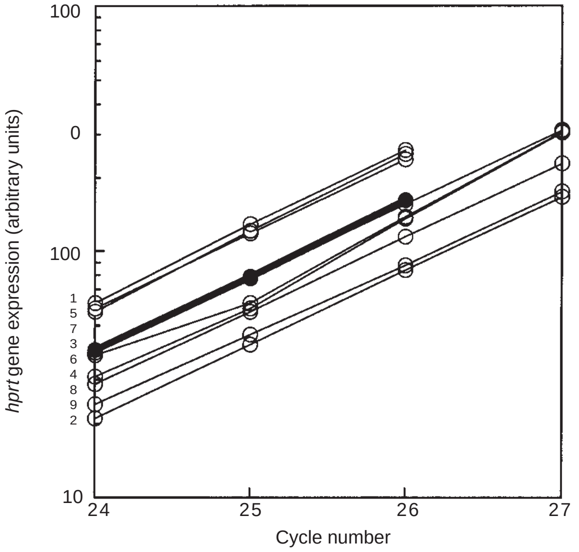

**Figure 4** Comparison of the linear amplification range of *top1* and *hprt* gene mRNA expression in the CRC samples. *hprt* mRNA expression (internal standard) as a function of the number of amplification cycles (1, X17LoVo; 2, LoVo; 3, HT29; 4, HT29A3; 5, TC1; 6,TC7fal; 7, TC71; 8, TC118m; 9, TC124m)

responded to high doses of CPT-11, but with only moderate responses for two of them (TC1 and TC124m). At low doses, one MSI$^-$ tumour (TC1), did not respond at all; the four other CRC xenografts (MSI$^+$ or MSI$^-$) were responders, and one (TC118m) was highly sensitive to CPT-11 with four CR of eight mice, but none were cured by CPT-11. Growth rate slopes of recurrences were similar to that of the controls. The sensitivity of CPT-11 treated tumours (TC118m) to subsequent treatment with CPT-11 was not diminished (data not shown).

To test the respective role of MSI and *p53* in the response to CPT-11, MSI$^+$ or MSI$^-$ tumour cell lines were used. The HT29 cell line is MSI$^-$/*mutp53* and resistant to CPT-11 at low dose, although at high dosage a mean growth inhibition of 66% could be obtained. HT29A3 is a tumorigenic subclone of HT29 cells transfected with *wtp53*. The transfected plasmid was located in chromosome 12 and dominant function of *wtp53* was attested to by arrest of the cell cycle when HT29A3 growth was inhibited by an inhibitor of pyrimidine synthesis. Transfection of *wtp53* in HT29 did not slow their growth in control mice and clearly increased their sensitivity to CPT-11, prolongating the survival of tumour-bearing mice, namely when treated at high dosage.

The LoVo cell line has a MSI$^+$ phenotype (Malkhosyan et al, 1996) and a *wtp53*. Its subclone, X17LoVo, was selected for high expression of *mutp53* which behaved like a dominant mutated p53 conferring a gain of function, as described in other models (Gualberto et al, 1998). γ-irradiation did not induce cell cycle arrest in G1/S (Pocard et al, 1996). Expression of *mutp53* in X17LoVo led to a reduction of sensitivity as compared to LoVo as shown by reduced growth delay and decreased growth inhibition, and shortening their survival time.

Taken together, these results show that among the three MSI$^+$ (TC7Fal, TC71 and LoVo) xenografts, all were sensitive at both dosages excepted LoVo which responded only at high dosage. Among the four MSI$^-$-CRC xenografts (TC1, TC118m, TC124m and HT29), one (HT29) was completely refractory to both dosages, and another (TC1) to low dosage only; the two others (TC118m and TC124m) displayed a high sensitivity whatever the dosage used. In both types (MSI$^+$ or MSI$^-$) mutation of p53 was also associated with a lack of responsiveness, which can be overcome by re-expression of wtp53.

In recent years, it has been proposed that genotypic alterations acquired during tumour progression may provide additional prognostic information. In colorectal cancers, a correlation between survival, 17p allelic losses and/or p53 mutations has been established (Hamelin et al, 1994). Tumour prognosis is determined either by intrinsic aggressiveness and/or the potential sensitivity to chemotherapy. The p53 point mutations seem to be crucial in these two parameters, at least in colorectal cancers; indeed p53-deficient tumours are more invasive and less responsive. Goh et al showed a shortened survival of *mutp53* CRC patients with post-operative therapies as compared to patients with CRCs with a functional p53 (Goh et al, 1995). This confirmed the study by Lowe showing that p53 plays a role in determining responsiveness to adjuvant chemotherapy (Lowe et al, 1994). Unresponsiveness of p53-deficient tumours has been ascribed to lack of apoptosis normally induced after G1/S-arrest following chemotherapy. Besides this clearly described programmed cell death, other alterations of cell cycle checkpoints in p53 deficient cells have been reported. By-passing of the G2/M arrest in p53-deficient cells after drug treatment drives them either to survive or die, probably depending on the accumulation of DNA lesions (Kastan et al, 1991; Ceraline et al, 1998), and this is independent of the induction of apoptosis (Attalla et al, 1997).

Different combinations of genetic alterations lead to different pathways of CRC progression. In a series of 87 CRCs, Olschwang et al found 41 MSI-/*mutp53* (47%), nine MSI$^+$/*wtp53* (10%), four MSI$^+$/*mutp53* (4.5%) and 33 MSI$^-$/*wtp53* (Olschwang et al, 1997). Fifteen per cent of CRCs were MSI$^+$ and more often associated with a *wtp53*. Fifty per cent had a p53 mutation and were rarely associated with a MSI phenotype. This inverse correlation was also found in CRC cell lines (Cottu et al, 1996). Sporadic MSI$^+$ CRCs and HNPCC have a better prognosis than cancers with MSI (Bubb et al, 1996; dos Santos et al, 1996; Vaurs and Bignon, 1997; Lukish et al, 1998) and reviewed by Arnheim and Shibata (1997). Their low progression potential is illustrated by the low frequency of MSI$^+$ liver metastases (Ishimaru et al, 1995).

Advances in adjuvant chemotherapy of colorectal cancers must address a complex series of factors including not only the availability of new effective compounds but also which subgroups of patients may benefit from them. Inhibitors of topoisomerase 1 are a new category of putatively potent anti-tumour agents. CPT-11 is a water soluble analogue of camptothecin, which is metabolized to

 © 2000 Cancer Research Campaign

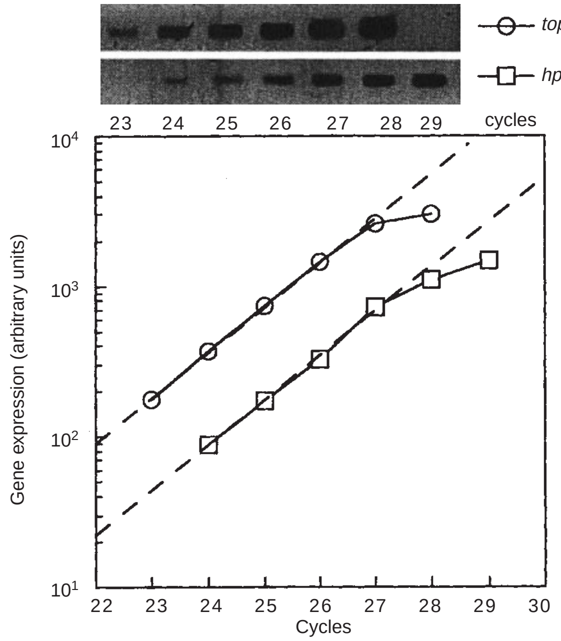

**Figure 5** Comparative mRNA expression of *top1* and *hprt*, as a function of the amplification cycle number. Upper part, UV-irradiated BET signals on agarose gel with an increasing number of cycles. Lower part, linear amplification of *hprt* and *top1* gene in the same sample, as reported on a diagram where the number of amplification cycles is plotted on the *x* axis and the gene expression, as evaluated by arbitrary units (corresponding to the fluorescent signal given by intercalating-cDNA irradiated BET on agarose gel) on the *y* axis

**Table 6** Relative gene expression in CRC xenografts. Ratios (× $10^{-2}$) of each gene expression to that of *HPRT* gene

| Samples | *top1* | *TP53* | *mdr1* | *fasR* |
|---|---|---|---|---|
| TC1 | 110 | 30 | 8 | 20 |
| TC124m | 105 | ND | 0.1 | 5 |
| TC118m | 290 | 300 | 0.2 | 70 |
| TC7fal | 140 | 120 | 4 | 100 |
| TC71 | 160 | 50 | 0.05 | 40 |
| LoVo | 90 | 30 | 2 | 60 |
| X17LoVo | 120 | 2700 | 0.6 | 60 |
| HT29 | 330 | 55 | 5 | 30 |
| HT29A3 | 400 | 75 | 7 | 20 |

ND, Not detected at 35 cycles.

an active compound, 7-ethyl-10-hydroxycamptothecin (SN38) β-glucuronide, by a carboxylesterase (Rivory et al, 1996). CPT-11 toxicity is a limiting factor (Armand et al, 1996). This contrasts with the tolerance in mice. Progresses in overcoming CPT-11 toxicity in humans should improve the clinical use of this compound (Takasuna et al, 1995, 1996). Nevertheless, even at the moderate doses tolerable by human hosts, top1 inhibitors are active agents in patients with metastatic colorectal cancers (Conti et al, 1996; Pazdur et al, 1997) and reviewed in Rougier and Bugat (1996); Rothenberg (1997).

CRC xenografts displayed a large range of sensitivity to CPT-11, from resistance to eradication. These differences were not due to different levels of top1, as evaluated by a semiquantitative method (Murphy et al, 1990), as already published (Goldwasser et al, 1995; van Ark-Otte et al, 1998). Jansen et al (1998) proposes a role for P-glycoprotein (encoded by *mdr1* gene) in lack of efficacy of numerous compounds, including CPT-11. Our study did not support such a role as no correlation was found between P-glycoprotein encoding gene expression (*MDR1*) and CPT-11 responses and, furthermore, a diversity of *mdr1* gene expression (a range of 80-fold) in our series of CRCs was shown.

The apoptotic process induced by drugs can involve the *fas* receptor pathway (Micheau et al, 1997) and it has been described that many CRCs express the fasR and initiate the death programme when activated (Meterissian et al, 1997; Tillman et al, 1998). Is CPT-11 activity mediated by activation of the fasR pathway in vivo, as shown in vitro in the HT29 cell line by Shao et al (1998)? In our series, HT29 tumours were the most CPT-11-resistant and they expressed a relatively low level of fasR, similar to that of HT29A3, its CPT-11-responsive counterpart, this excluding such correlation.

It was published that sensitivity to CPT-11 depends on the accumulation and stability of cleavable complexes, and this could be a parameter explaining the inter tumour differences. Cleavable complex levels were measured in vivo after camptothecin treatment in colon carcinoma cell lines by Goldwasser et al who showed a direct correlation between levels of cleavable complexes and cytotoxicity (Goldwasser et al, 1995). This has not been tested in vitro in our study as the methodology is in the process of being developed for in vivo samples but is not presently available.

In conclusion, our results indicate that CRCs are good candidates for CPT-11 chemotherapy whatever their MSI phenotype as long as they have a wild-type p53, whereas some, but probably not all mutations of p53, have the propensity to overcome CPT-11 sensitivity.

### ACKNOWLEDGEMENTS

We are grateful to V Bordier and C Alberti for excellent technical assistance in animal experimentation. We thank D Rouillard, laboratoire de Physiopathology, Section Médicale et Hospitalière de l'Institut Curie, for her help in cell cycle analysis. We thank Dr S Agrawal for reviewing the English used in this report. We are grateful to Rhône Poulenc Rorer for furnishing the necessary amounts of Irinotecan (CPT-11). RA Bras-Gonçalves was supported by a fellowship from the Luxembourg Government R/D BFR 95/035. P Soulié was supported by the Fondation pour la Recherche Médicale, Paris, France.

### REFERENCES

Armand JP, Ducreux M, Mahjoubi M, Abigerges D, Bugat R, Chabot G, Herait P, de Forni M and Rougier P (1995) CPT-11 (irinotecan) in the treatment of colorectal cancer. *Eur J Cancer* **31A**: 1283–1287

Armand JP, Extra YM, Catimel G, Abigerges D, Marty M and Clavel M (1996) Rationale for the dosage and schedule of CPT-11 (irinotecan) selected for phase II studies, as determined by European phase I studies. *Ann Oncol* **7**: 837–842

Arnheim N and Shibata D (1997) DNA mismatch repair in mammals: role in disease and meiosis. *Curr Opin Gen Dev* **7**: 364–370

Attalla H, Makela TP, Wahala K, Rasku S, Andersson LC and Adlercreutz H (1997) 2,6-Bis((3,4-dihydroxyphenyl)-methylene)cyclohexanone (BDHPC)-induced apoptosis and p53-independent growth inhibition: synergism with genistein. *Biochem Biophys Res Commun* **239**: 467–472

© 2000 Cancer Research Campaign 

Bubb VJ, Curtis LJ, Cunningham C, Dunlop MG, Carothers AD, Morris RG, White S, Bird CC and Wyllie AH (1996) Microsatellite instability and the role of hMSH2 in sporadic colorectal cancer. *Oncogene* **12**: 2641–2649

Casares S, Ionov Y, Ge HY, Stanbridge E and Perucho M (1995) The microsatellite mutator phenotype of colon cancer cells is often recessive. *Oncogene* **11**: 2303–2310

Ceraline J, Deplanque G, Duclos B, Limacher JM, Hajri A, Noel F, Orvain C, Frebourg T, Klein-Soyer C and Bergerat JP (1998) Inactivation of p53 in normal human cells increases G2/M arrest and sensitivity to DNA-damaging agents. *Int J Cancer* **75**: 432–438

Conti JA, Kemeny NE, Saltz LB, Huang Y, Tong WP, Chou TC, Sun M, Pulliam S and Gonzalez C (1996) Irinotecan is an active agent in untreated patients with metastatic colorectal cancer. *J Clin Oncol* **14**: 709–715

Cottu PH, Muzeau F, Estreicher A, Flejou JF, Iggo R, Thomas G and Hamelin R (1996) Inverse correlation between RER+ status and p53 mutation in colorectal cancer cell lines. *Oncogene* **13**: 2727–2730

de Gramont A, Bosset JF, Milan C, Rougier P, Bouche O, Etienne PL, Morvan F, Louvet C, Guillot T, Francois E and Bedenne L (1997) Randomized trial comparing monthly low-dose leucovorin and fluorouracil bolus with bimonthly high-dose leucovorin and fluorouracil bolus plus continuous infusion for advanced colorectal cancer: a French intergroup study. *J Clin Oncol* **15**: 808–815

dos Santos NR, Seruca R, Constancia M, Seixas M and Sobrinho-Simoes M (1996) Microsatellite instability at multiple loci in gastric carcinoma: clinicopathologic implications and prognosis. *Gastroenterology* **110**: 38–44

el-Mahdani N, Vaillant JC, Guiguet M, Prevot S, Bertrand V, Bernard C, Parc R, Bereziat G and Hermelin B (1997) Overexpression of p53 mRNA in colorectal cancer and its relationship to p53 gene mutation. *Br J Cancer* **75**: 528–536

Fan S, el-Deiry WS, Bae I, Freeman J, Jondle D, Bhatia K, Fornace AJ, Jr, Magrath I, Kohn KW and O'Connor PM (1994) p53 gene mutations are associated with decreased sensitivity of human lymphoma cells to DNA damaging agents. *Cancer Res* **54**: 5824–5830

Fujiwara T, Grimm EA, Mukhopadhyay T, Zhang WW, Owen-Schaub LB and Roth JA (1994) Induction of chemosensitivity in human lung cancer cells in vivo by adenovirus-mediated transfer of the wild-type p53 gene. *Cancer Res* **54**: 2287–2291

Goh HS, Yao J and Smith DR (1995) p53 point mutation and survival in colorectal cancer patients. *Cancer Res* **55**: 5217–5221

Goldberg RM, Hatfield AK, Kahn M, Sargent DJ, Knost JA, O'Connell MJ, Krook JE, Maillard JA, Wiesenfeld M, Schaefer PL, Tirona MT and Moertel CG (1997) Prospectively randomized North Central Cancer Treatment Group trial of intensive-course fluorouracil combined with the 1-isomer of intravenous leucovorin, oral leucovorin, or intravenous leucovorin for the treatment of advanced colorectal cancer. *J Clin Oncol* **15**: 3320–3329

Goldwasser F, Bae I, Valenti M, Torres K and Pommier Y (1995) Topoisomerase I-related parameters and camptothecin activity in the colon carcinoma cell lines from the National Cancer Institute anticancer screen. *Cancer Res* **55**: 2116–2121

Gottesman MM, Pastan I and Ambudkar SV (1996) P-glycoprotein and multidrug resistance. *Curr Opin Genet Dev* **6**: 610–617

Gualberto A, Aldape K, Kozakiewicz K and Tlsty TD (1998) An oncogenic form of p53 confers a dominant, gain-of-function phenotype that disrupts spindle checkpoint control. *Proc Natl Acad Sci USA* **95**: 5166–5171

Hamelin R, Jego N, Laurent-Puig P, Vidaud M and Thomas G (1993) Efficient screening of p53 mutations by denaturing gradient gel electrophoresis in colorectal tumors. *Oncogene* **8**: 2213–2220

Hamelin R, Laurent-Puig P, Olschwang S, Jego N, Asselain B, Remvikos Y, Girodet J, Salmon RJ and Thomas G (1994) Association of p53 mutations with short survival in colorectal cancer [see comments]. *Gastroenterology* **106**: 42–48

Hoang JM, Cottu PH, Thuille B, Salmon RJ, Thomas G and Hamelin R (1997) BAT-26, an indicator of the replication error phenotype in colorectal cancers and cell lines. *Cancer Res* **57**: 300–303

Houghton PJ, Cheshire PJ, Hallman II JD, Lutz L, Friedman HS, Danks MK and Houghton JA (1995) Efficacy of topoisomerase I inhibitors, topotecan and irinotecan, administered at low dose levels in protracted schedules to mice bearing xenografts of human tumors. *Cancer Chemoth Pharm* **36**: 393–403

Ishimaru G, Adachi J, Shiseki M, Yamaguchi N, Muto T and Yokota J (1995) Microsatellite instability in primary and metastatic colorectal cancers. *Int J Cancer* **64**: 153–157

Jansen WJ, Hulscher TM, van Ark-Otte J, Giaccone G, Pinedo HM and Boven E (1998) CPT-11 sensitivity in relation to the expression of P170-glycoprotein and multidrug resistance-associated protein. *Br J Cancer* **77**: 359–365

Kaneda N, Nagata H, Furuta T and Yokokura T (1990) Metabolism and pharmacokinetics of the camptothecin analogue CPT-11 in the mouse. *Cancer Res* **50**: 1715–1720

Kastan MB, Onyekwere O, Sidransky D, Vogelstein B and Craig RW (1991) Participation of p53 protein in the cellular response to DNA damage. *Cancer Res* **51**: 6304–6311

Kjeldsen E, Svejstrup JQ, Gromova II, Alsner J and Westergaard O (1992) Camptothecin inhibits both the cleavage and religation reactions of eukaryotic DNA topoisomerase I. *J Mol Biol* **228**: 1025–1030

Köhler T, Lassner D, Rost A-K, Thamm B, Pustowoit B and Remke H (1995) *Quantitation of mRNA by Polymerase Chain Reaction, Non-radioactive PCR Methods*. Springer-Verlag: Berlin

Kunimoto T, Nitta K, Tanaka T, Uehara N, Baba H, Takeuchi M, Yokokura T, Sawada S, Miyasaka T and Mutai M (1987) Antitumor activity of 7-ethyl-10-[4-(1-piperidino)-1-piperidino]carbonyloxy-camptothecin, a novel water-soluble derivative of camptothecin, against murine tumors. *Cancer Res* **47**: 5944–5947

Laurence J, Mitra D, Steiner M, Lynch D, Siegal F and Staiano-Coico L (1996) Apoptotic depletion of CD4+ T cells in idiopathic CD4+ T lymphocytopenia. *Am Soc Clin Invest* **97**: 672–680

Leteurtre F, Fesen M, Kohlhagen G, Kohn KW and Pommier Y (1993) Specific interaction of camptothecin, a topoisomerase I inhibitor, with guanine residues of DNA detected by photoactivation at 365 nm. *Biochemistry* **32**: 8955–8962

Lowe SW, Ruley HE, Jacks T and Housman DE (1993) p53-dependent apoptosis modulates the cytotoxicity of anticancer agents. *Cell* **74**: 957–967

Lowe SW, Bodis S, McClatchey A, Remington L, Ruley HE, Fisher DE, Housman DE and Jacks T (1994) p53 status and the efficacy of cancer therapy in vivo. *Science* **266**: 807–810

Lukish JR, Muro K, DeNobile J, Katz R, Williams J, Cruess DF, Drucker W, Kirsch I and Hamilton SR (1998) Prognostic significance of DNA replication errors in young patients with colorectal cancer. *Ann Surg* **227**: 51–56

McDonald AC and Brown R (1998) Induction of p53-dependent and p53-independent cellular responses by topoisomerase 1 inhibitors. *Br J Cancer* **78**: 745–751

Malkhosyan S, McCarty A, Sawai H and Perucho M (1996) Differences in the spectrum of spontaneous mutations in the hprt gene between tumor cells of the microsatellite mutator phenotype. *Mutat Res* **316**: 249–259

Meterissian SH, Kontogiannea M, Po J, Jensen G and Ferdinand B (1997) Apoptosis induced in human colorectal carcinoma by anti-Fas antibody. *Ann Surg Oncol* **4**: 169–175

Micheau O, Solary E, Hammann A, Martin F and Dimanche-Boitrel MT (1997) Sensitization of cancer cells treated with cytotoxic drugs to fas-mediated cytotoxicity. *J Natl Cancer* **189**: 783–789

Moertel CG, Fleming TR, Macdonald JS, Haller DG, Laurie JA, Goodman PJ, Ungerleider JS, Emerson WA, Tormey DC, Glick JH and et al (1990) Levamisole and fluorouracil for adjuvant therapy of resected colon carcinoma [see comments]. *N Engl J Med* **322**: 352–358

Murphy LD, Herzog CE, Rudick JB, Fojo AT and Bates SE (1990) Use of the polymerase chain reaction in the quantitation of mdr-1 gene expression. *Biochemistry* **29**: 10351–10356

Olschwang S, Hamelin R, Laurent-Puig P, Thuille B, De Rycke Y, Li YJ, Muzeau F, Girodet J, Salmon RJ and Thomas G (1997) Alternative genetic pathways in colorectal carcinogenesis. *Proc Natl Acad Sci USA* **94**: 12122–12127

Pazdur R, Diaz-Canton E, Ballard WP, Bradof JE, Graham S, Arbuck SG, Abbruzzese JL and Winn R (1997) Phase II trial of 9-aminocamptothecin administered as a 72-hour continuous infusion in metastatic colorectal carcinoma. *J Clin Oncol* **15**: 2905–2909

Pocard M, Chevillard S, Villaudy J, Poupon MF, Dutrillaux B and Remvikos Y (1996) Different p53 mutations produce distinct effects on the ability of colon carcinoma cells to become blocked at the G1/S boundary after irradiation. *Oncogene* **12**: 875–882

Pommier Y (1996) Eukaryotic DNA topoisomerase I: genome gatekeeper and its intruders, camptothecins. *Semin Oncol* **23**: 3–10

Rivory LP, Riou JF, Haaz MC, Sable S, Vuilhorgne M, Commercon A, Pond SM and Robert J (1996) Identification and properties of a major plasma metabolite of irinotecan (CPT-11) isolated from the plasma of patients. *Cancer Res* **56**: 3689–3694

Rothenberg ML (1997) Topoisomerase I inhibitors: review and update. *Ann Oncol* **8**: 837–855

Rougier P and Bugat R (1996) CPT-11 in the treatment of colorectal cancer: clinical efficacy and safety profile. *Semin Oncol* **23**: 34–41

Rougier P, Bugat R, Douillard JY, Culine S, Suc E, Brunet P, Becouarn Y, Ychou M, Marty M, Extra JM, Bonneterre J, Adenis A, Seitz JF, Ganem G, Namer M, Conroy T, Negrier S, Merrouche Y, Burki F, Mousseau M, Herait P and Mahjoubi M (1997) Phase II study of irinotecan in the treatment of advanced colorectal cancer in chemotherapy-naive patients and patients pretreated with fluorouracil-based chemotherapy. *J Clin Oncol* **15**: 251–260

© 2000 Cancer Research Campaign

Shao RG, Cao CX, Nieves-Neira W, Pilon AA and Strumberg D (1998) Camptothecin induced apoptosis in human colon carcinoma p53 mutant HT29 cells: role of apocrine signaling through Fas and cytokine pathways. *Proc Am Assoc Cancer Res* **39**: 178

Soulie P, Poupon MF, Remvikos Y, Dutrillaux B and Muleris M (1999) Distinct chromosomal alterations associated with TP53 status of LoVo cells under PALA selective pressure: a parallel with cytogenetic pathways of colorectal cancers. *Oncogene* **18**: 775–781

Strater J, Wellisch I, Riedl S, Walczak H, Koretz K, Tandara A, Krammer PH and Moller P (1997) CD95 (APO-1/Fas)-mediated apoptosis in colon epithelial cells: a possible role in ulcerative colitis. *Gastroenterology* **113**: 160–167

Takasuna K, Kasai Y, Kitano Y, Mori K, Kobayashi R, Hagiwara T, Kakihata K, Hirohashi M, Nomura M, Nagai E and et al (1995) Protective effects of kampo medicines and baicalin against intestinal toxicity of a new anticancer camptothecin derivative, irinotecan hydrochloride (CPT-11), in rats. *JPN J Cancer Res* **86**: 978–984

Takasuna K, Hagiwara T, Hirohashi M, Kato M, Nomura M, Nagai E, Yokoi T and Kamataki T (1996) Involvement of beta-glucuronidase in intestinal microflora in the intestinal toxicity of the antitumor camptothecin derivative irinotecan hydrochloride (CPT-11) in rats. *Cancer Res* **56**: 3752–3757

Tanizawa A, Fujimori A, Fujimori Y and Pommier Y (1994) Comparison of topoisomerase I inhibition, DNA damage, and cytotoxicity of camptothecin derivatives presently in clinical trials. *J Natl Cancer I* **86**: 836–842

Tanizawa A, Kohn KW, Kohlhagen G, Leteurtre F and Pommier Y (1995) Differential stabilization of eukaryotic DNA topoisomerase I cleavable complexes by camptothecin derivatives. *Biochemistry* **34**: 7200–7206

Tillman DM, Harwood FG, Gibson AA and Houghton JA (1998) Expression of genes that regulate Fas signalling and Fas-mediated apoptosis in colon carcinoma cells. *Cell Death Differentiation* **5**: 450–457

Vamvakas S, Vock EH and Lutz WK (1997) On the role of DNA double-strand breaks in toxicity and carcinogenesis. *Crit Rev Toxicol* **27**: 155–174

van Ark-Otte J, Kedde MA, van der Vijgh WJ, Dingemans AM, Jansen WJ, Pinedo HM, Boven E and Giaccone G (1998) Determinants of CPT-11 and SN-38 activities in human lung cancer cells. *Brit J Cancer* **77**: 2171–2176

Vaurs C and Bignon YJ (1997) What's new about microsatellite instability significance in human carcinogenesis? *Bull Cancer* **84**: 1061–1071

Wall ME, Wani MC, Cooke CE, Palmer KH, McPhail AT and Slim GA (1966) *J Am Chem Soc* **88**: 3888–3890

Wilke H (1996) *Semin Oncol* **23**: 1–2

Zhou XP, Hoang JM, Li YJ, Seruca R, Carneiro F, Sobrinho-Simoes M, Lothe RA, Gleeson CM, Russell SE, Muzeau F, Flejou JF, Hoang-Xuan K, Lidereau R, Thomas G and Hamelin R (1998) Determination of the replication error phenotype in human tumors without the requirement for matching normal DNA by analysis of mononucleotide repeat microsatellites. *Genes Chromosomes Cancer* **21**: 101–107

*© 2000 Cancer Research Campaign*
